# Reference gene validation for the relative quantification of cannabinoid receptor expression in human odontoblasts via quantitative polymerase chain reaction

**DOI:** 10.1016/j.jobcr.2022.09.006

**Published:** 2022-09-10

**Authors:** Laura M. Navarro-Saiz, Lilia J. Bernal-Cepeda, Felipe García-Jiménez, Deisy Abril, Jaime E. Castellanos

**Affiliations:** a Grupo de Investigación Básica y Aplicada en Odontología, Universidad Nacional de Colombia, Bogotá, 111321, Colombia; b Bacterial Molecular Genetics Laboratory, Universidad El Bosque, Bogotá, 110121, Colombia

**Keywords:** Reference gene, Odontoblast, RT-qPCR, Cannabinoid receptor 1, Cannabinoid receptor 2, CB, cannabinoid, GAPDH, glyceraldehyde‐3‐phosphate dehydrogenase, OLCs, odontoblast-like cells, RT-qPCR, reverse transcription-quantitative polymerase chain reaction

## Abstract

**Objective:**

The aim of this study was to identify and validate the reference genes in cultured human odontoblasts to quantify their cannabinoid receptor transcripts.

**Methods:**

The most stably transcribed genes in cultured human odontoblast cells were identified using the RefGenes tool and were selected for real-time polymerase chain reaction (PCR) amplification. Human odontoblast cells were differentiated from mesenchymal stem cells using a transforming growth factor-β-supplemented differentiation medium, and total RNA was purified. Reverse transcription-quantitative PCR and relative quantification analyses were performed using the Schefe's method. The relative expression dataset was analyzed to select the most stable genes.

**Results:**

The analysis showed that the transcripts of cholinergic receptor nicotinic beta 2 subunit, LIM homeobox transcription factor 1 beta, and family with sequence similarity 223 member B presented the lowest standard deviation (SD) in expression (SD: 0.2, 0.17, and 0.16, respectively). These genes showed similar expression levels as the target genes (cannabinoid receptors). Significant differences were found in the relative expression levels of cannabinoid receptors using the selected genes compared to those calculated using beta actin transcripts as references (p < 0.05).

**Conclusions:**

The strategy reported here for searching and verifying new reference genes will aid in the accurate and reliable expression of cannabinoid receptors in human odontoblast cells.

## Highlights

• Cannabinoid receptors play an essential role regulating pain and inflammation.
• Reference gene validation is needed to improve the accuracy of RT qPCR results.
• Bioinformatic and experimental techniques are suitable to find the most stable gen.
• A significant difference in expression is observed normalizing with CRN or β-actin.

## Graphical abstract

**Figure:**
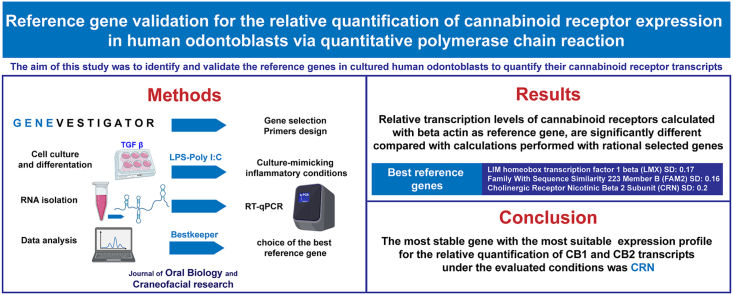

## 1 Introduction

Through relative quantification, the evaluation of gene expression using reverse transcription-quantitative polymerase chain reaction (RT-qPCR) facilitates the determination of transcriptional changes in the gene of interest compared to its basal levels or those in the untreated samples. This value expresses the ratio of the number of transcripts of the gene of interest to the number of reference gene messengers.^1^ Therefore, choosing a suitable quantification method and appropriate reference genes is crucial to determine the expression change in a more accurate and precise manner, and subsequently, to obtain more reliable results.^2^

The Minimum Information for Publication of Quantitative Real-Time PCR Experiments guidelines outline the basic rules for designing and publishing qPCR experiments.^3^ The use of two or more reference genes is recommended, but they should be validated to ensure stable expression in the treatment groups for the given experimental setting and sample set.

Due to the importance of the reference genes or materials used for standardization, any assessment of the validity of an RT-qPCR experiment must verify the suitability of the relative quantification reference, and its utility must be empirically validated for particular tissues, cell types, and experimental designs.^4^^,^^5^ Using inappropriate reference genes for quantification can lead to misleading results.

Since normalization involves calculating the ratio of the mRNA levels of genes of interest to those of reference genes, the latter should be stably expressed, and their abundance should show a strong correlation with the total mRNA levels present in the samples.^6^ In addition, transcript levels of genes of interest and reference genes should be similar to ensure that all transcripts are subject to the same kinetic interactions during qPCR processing.^7^

Normalization against a single reference gene is not acceptable unless robust evidence is presented to confirm its invariant expression across different conditions. Thus, at least two reference genes should be employed, and the geometric mean of their expression levels should be used for normalization.^2^^,^^6^^,^^7^ Hence, the optimal number and choice of reference genes should always be experimentally determined.

Hruz et al. (2011) tested two hypotheses for using reference genes; the first hypothesis is about non-generality that states that no gene is stably expressed as all are regulated to some extent, so there are no genes with universally stable expression under any condition. The second one is about biological context specificity that states for each condition, there is a subset of genes showing low expression variation. Therefore, the reference genes should not be used without validation. However, a systematic review showed that when validation is performed for the selected genes, common reference genes, such as actin beta (*β-actin)*, glyceraldehyde‐3‐phosphate dehydrogenase (*GAPDH*), and *18S*, are selected in approximately 50% of all cases.^7^

Since no reference gene validation has been reported for human odontoblast-like cells (OLCs) that are differentiated from dental pulp mesenchymal stem cells, the objective of the present work was to describe and perform a test for the identification, selection, and validation of reference genes to perform relative quantification of the cannabinoid (CB)-1 and CB2 receptor transcript expression levels in these cells.

## 2 Materials and methods

### 2.1 Gene selection

The RefGenes tool of Genevestigator (http://www.genevestigator.com/) was used to select the most stable genes, where large microarray datasets were compiled for a specific condition or subset of conditions. This tool aid in the identification of genes that exhibit minimal variation in expression on a chosen set of arrays for a particular biological context. In this case, matrices corresponding to human osteoblasts, which show a mineralizing phenotype similar to that of OLCs, were chosen since no report was found for the latter. Dental pulp matrices were used in this study. From the list of 20 proposed genes, four genes were chosen for OLCs and four for dental pulp based on minor standard deviation (SD) and ease of access to sequences.

### 2.2 Primers design

For primer design, it was necessary to analyze the eight candidate genes defined by Genevestigator. Four candidate sequences for validation corresponded to non-protein-coding genes; these sequences were considered as PCR detects transcripts similar to rRNA. Genes with expression levels similar to that of the target gene, with little variation (low SD), were selected.

Information about the candidate genes was obtained using GeneCards (https://www.genecards.org), an open access database where the size of each gene, its complete and alternative names, its function within the organism and/or specific cell, and its location in the chromosome can be found. It also gives access to the DNA sequence of the gene as well as information on exons. Through GeneCards, permits were obtained from the National Center for Biotechnology Information GenBank database, where the nucleotide sequence of each gene was obtained. Based on the genomic coordinates of the genes, exons of adequate size were selected, with information supplied by the GeneLoc program, annexed to GeneCards. Then, using the FASTA format, primers were designed from two or three regions of sequences obtained from GenBank using the Primer-Basic Local Alignment Search Tool (BLAST) tool. Primer specificity was confirmed by entering the sequences into BLAST (https://blast.ncbi.nlm.nih.gov/Blast.cgi). The results are listed, including the names of the sequences with similarities, species of origin, identity score, coverage and percentage identity, and sequence accession codes.

The primers flanked an expected product size between 150 and 170 bp to ensure adequate reaction efficiency and a sufficient amplicon size for the design of real-time hydrolysis probes. The melting temperature parameters were as follows: minimum, optimal, and maximum temperatures of 57, 60, and 63 °C, respectively, and the maximum Tm difference between primer pairs was set at 3 °C. At the same time, the parameters for primer pair specificity in amplification were set, enabling automatic search for primers specific to the selected template, ignoring templates that had six or more discrepancies in complementarity with the primer sequence, and including primers that had at least two differences with unspecific targets. The species specificity of the primers was limited to *Homo sapiens*.

### 2.3 Cell culture

Human odontoblast cells were differentiated from healthy third molar dental pulp mesenchymal stem cells using a differentiation medium and transforming growth factor (TGF)-beta, as previously described.^8^ Around 25,000 cells were seeded in three 6-well plates with Dulbecco's Modified Eagle Culture Medium (Hyclone, Thermo Scientific, Bremen, Germany) supplemented with 10% fetal bovine serum (Gibco; Thermo Fisher Scientific), 1% antibiotic (100 U/mL penicillin +100 μg/mL streptomycin), and 10 ng/mL of TGF-β1 (Abcam, Cambridge, MA, USA) and incubated for 24 h at 37 °C in a humidified atmosphere with 5% CO_2_ until 70% confluence was reached. In addition, odontoblast culture-mimicking inflammatory conditions were maintained via 24 h stimulation of the cells in some wells with 2 μg/mL of *Escherichia coli* lipopolysaccharide (LPS) (Sigma-Aldrich, St. Louis, MO) or 40 μg/mL of Poly-I:C (InvivoGen, St. Diego, CA) to simulate bacterial or viral infection, respectively; unstimulated cells were used as a control. Cells were harvested, and RNA isolation was performed using the TRIzol reagent (Ambion).

### 2.4 Gene validation

RT-qPCR was performed using SYBR Green (Luna® Universal One RT-qPCR Kit; New England BioLabs, USA) and a CFX96 real-time thermocycler to determine the variations in expression levels under different conditions. The amplification conditions were as follows: retrotranscription for 10 min at 55 °C, denaturation for 3 min at 95 °C, 40 amplification cycles at a denaturation temperature of 95 °C for 15 s, and an annealing temperature of 60 °C for 30 s. Finally, a melting curve was generated to confirm the specificity of the amplified products. In addition to the chosen genes, three commonly used reference genes (*GAPDH*, *β-actin*, and *18S* rRNA), two genes as markers of the odontoblastic phenotype (dentin sialophosphoprotein and dentin matrix acidic phosphoprotein 1), and two target genes (*CB1* and *CB2*) were amplified (Table 1).

**Table 1:** Genes and primers used in gene validation.

Gene/RefSeq	Gene name	Function	Forward primer 5′- 3′	Reverse primer 5′- 3′	Code	Amplicon (bp)	Reference
**ODONTOBLAST**							
**LBX1-AS1** NC_000010.11	LBX1 Antisense RNA 1	RNA Gene	CCTTTGGAAACCAGCCCACC	AAGAGGGGTACAAGAGGCAAG	LBX	150	This work
**LRRC2-AS1** NC_000003.12	LRRC2 Antisense RNA 1	RNA Gene	CCTTTGGAAACCAGCCCACC	CTGCCCACACTGCTCAAATAC	LRR	173	This work
**ADAMTS7** NC_000015.10	ADAM Metallopeptidase With Thrombospondin Type 1 Motif 7	Regulate vascular smooth muscle cell (VSMC) migration	CACAGTGAGACCAGGGATGTC	TAGCAGGACCCTGGAAAGGAG	ADA	153	This work
**CHRNB2** NC_000001.11	Cholinergic Receptor Nicotinic Beta 2 Subunit	Ligand-gated ion channels	CAATGCTGACGGCATGTACGA	CACGAACGGAACTTCATGGTG	CRN	165	21, 22
**DENTAL PULP**							
**LINC02097** NC_000017.11	Long Intergenic Non-Protein Coding RNA 2097	RNA Gene	GCTAGATAAGACTGGAAGACAGCA	TTGGCTTGAGATGCCTTGTT	LINC	157	This work
**FAM223B** NC_000023.11	Family With Sequence Similarity 223 Member B	RNA Gene	AGCAGAGTACCGACGAAAGG	ACGTTTTGAGCCCTTATTGGGA	FAM2	155	This work
**CHRM4** NC_000011.10	Cholinergic Receptor Muscarinic 4	Binding of acetylcholine	TCACCAAGCCTCTCACCTACCC	TCCGCTTACCCACCACAAACTG	CRM	135	^23^
**LMX1B** NC_000009.12	LIM Homeobox Transcription Factor 1 Beta	Transcription factor	GAGAAGATCGCCCCCACC	TTCTCCTTCTCGTAGTCACCCT	LMX	161	This work
**COMMONLY USED REFERENCE GENES**							
**ACTB** NC_000007.14	Actin Beta	Cell motility, structure, integrity, and intercellular signaling	GGATGCAGAAGGAGATCACTG	CGATCCACACGGAGTACTTG	B act	90	24, 25
**GAPDH** NC_000012.12	Glyceraldehyde-3-Phosphate Dehydrogenase	Perform mechanistically distinct functions	CACTAGGCGCTCACTGTTCTC	AAATCCGTTGACTCCGACCT	GAPDH	90	26, 27
**RPS18** NC_000006.12	Ribosomal Protein S18	Catalyze protein synthesis			18S	186	
**ODONTOBLASTPHENOTYPE MARKERS**							
**DSPP** NC_000004.12	Dentin Sialophosphoprotein	Dentin extracellular matrix	AGAAGGACCTGGCCAAAAAT	TCTCCTCGGCTACTGCTGTT	DSPP	201	28, 29
**DMP1** NC_000004.12	Dentin Matrix Acidic Phosphoprotein 1	Dentin extracellular matrix	GAACAGTGCAGGCATGAAATC	CTGAGATGCGAGACTTCCTAAA	DMP1	128	^30^
**TARGET GENES**							
**CNR1** NC_000006.12	Cannabinoid Receptor 1	GPCR receptor	GGTTAGCAAGATACACTCAAGCATGA	CTGGAAAAAGGCCCAACAAG	CB1	109	^31^
**CNR2** NC_000001.11	Cannabinoid Receptor 2	GPCR receptor	GACACGGACCCCTTTTTGCT	CCTCGTGGCCCTACCTATCC	CB2	103	31, 32, 33, 34

The PCR efficiency was calculated using LinRegPCR (Academic Medical Center, AMC, Amsterdam, Netherlands). Relative quantification was performed for CB1 and CB2 receptor transcripts using the Schefe's method^9^ taking unstimulated cells as a control sample, and normalizing them with the best reference gene (obtained using the Bestkeeper software https://www.gene-quantification.de), with lowest SD and expression levels similar to target genes as the selection criteria.

### 2.4 Data analysis

The data were organized in spreadsheets in Excel (Microsoft Office 2010). The quantification cycle (Cq) values and efficiencies of the reference and target genes were entered into the BestKeeper Excel template to select the best reference genes. This index calculates the geometric mean of the genes expressed with a SD of less than 1 and Pearson's correlation coefficient. Plots were generated using GraphPad Prism 7.0 software (GraphPad Software, San Diego, CA, USA). All experiments were performed in duplicate and repeated thrice (n = 12) per condition. One-way analysis of variance was used to determine the p-value <0.05.

## 3 Results

Eleven genes were selected as candidates for the analysis as reference genes. Table 1 shows a list of genes and primers designed to perform validation in human odontoblasts. The primers amplified all variants of each gene and were specific to *Homo sapiens*.

**Fig. 1:**
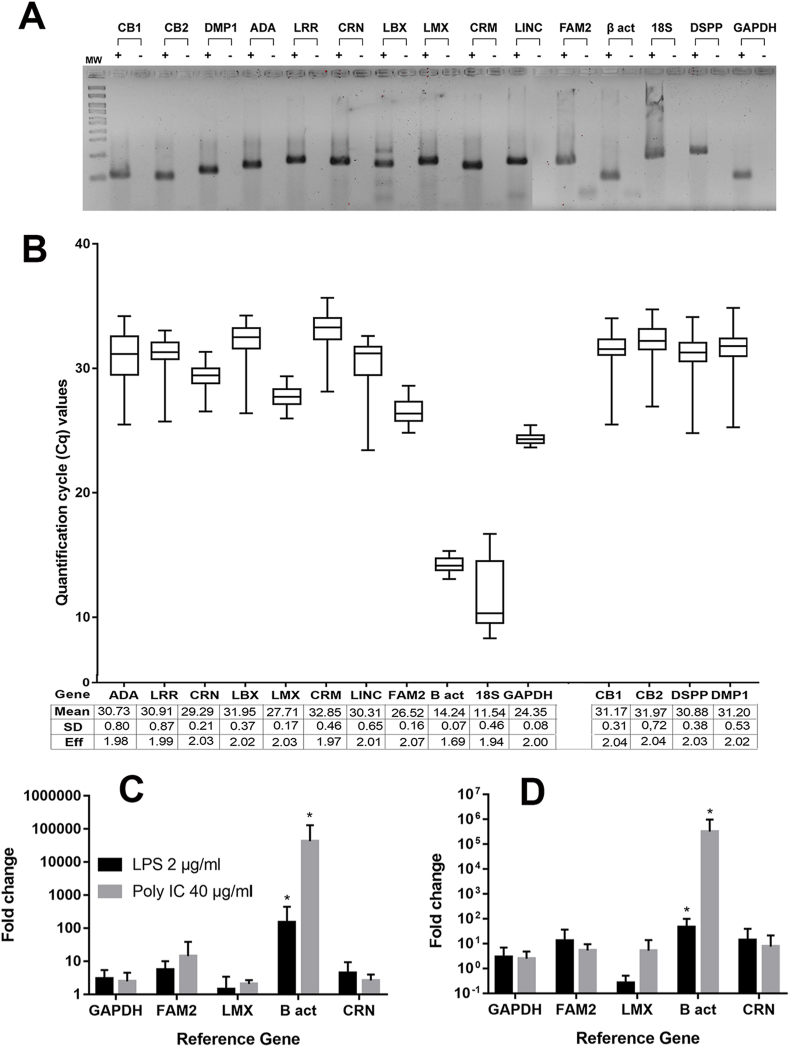
A. 2% agarose gel with RT-qPCR amplification products for the chosen reference genes and their respective negative controls. B. Expression levels of evaluated genes. Expression profiles of 11 candidate reference genes, two odontoblast marker genes, and two target genes, and their absolute quantification cycle (Cq) values in different conditions, including 2 μg/mL of lipopolysaccharide (LPS), 40 μg/mL of Poly-I:C, and unstimulated cells. Mean, standard deviation (SD), and efficiency (E) of each amplified gene. C. Relative quantification of cannabinoid (CB)-1 receptors in human odontoblasts stimulated with LPS and Poly-I:C using different reference genes. D. Relative quantification of cannabinoid (CB)-2 receptors in human odontoblasts stimulated with LPS and Poly-I:C using different reference genes.

Primer specificity was confirmed by separating the RT-qPCR amplification products on 2% agarose gel. All primers generated specific products, because a single band was observed at the expected amplicon size and their respective negative controls (Fig. 1A). The Cq values obtained by RT-qPCR were used to provide an overview of the expression levels of the candidate genes in all samples. The mean Cq values of the reference genes were between 11 and 32.

The *18S* subunit was the most abundantly expressed gene, with the lowest mean Cq value (11.54) and SD between 0.07 and 0.8, while *GAPDH* showed the lowest variation in expression (0.08). The amplification efficiency of all genes ranged between 1.94 and 2.07, while that of *β-actin* was the lowest (1.69) (Fig. 1B). Based on these data, cholinergic receptor nicotinic beta 2 subunit (*CHRNB2*/*CRN*), family with sequence similarity 223 member B (*FAM223B/FAM2*), and LIM homeobox transcription factor 1 beta (*LMX1B/LMX*) were chosen as possible reference genes as they had the lowest SDs and expression levels similar to those of the target genes.

Gene expression stability analysis was performed using the BestKeeper index, where the geometric mean, SD, correlation coefficient, and p-value were calculated. The most stable reference genes were *CRN*, *LMX*, and *FAM2*, as they had a higher correlation coefficient (r) among genes with SD less than 1 and an x-fold less than 2 (Table 2).

**Table 2:** The geometric means (GMs), standard deviation (SDs), coefficient of correlation (r), p-value, and power of housekeeping genes (HKGs) showing the correlation between each gene and the BestKeeper index.

	B act	GAPDH	LMX^a^	FAM2^a^	CNR^a^	CMR	LINC	LRR	LBX	ADA
**Cq geometric mean (GM)**	14.23	24.34	27.70	26.51	29.27	32.81	30.23	30.88	31.89	30.66
**Standard Deviation (SD)**	0.53	0.38	0.63	0.78	0.72	1.20	1.68	1.04	1.36	1.68
**Coefficient of Correlation (*r*)**	0.75	0.67	0.88	0.76	0.93	0.94	0.94	0.90	0.94	0.76
**p-value**	0.002	0.001	0.001	0.001	0.001	0.001	0.001	0.001	0.001	0.001
**Power of HKG (x-fold)**	1.26	1.22	1.58	1.62	1.82	3.20	2.64	3.68	2.72	2.36

a Most stable genes.

The number of fold changes in CB1 and CB2 expression levels obtained after the use of β-act was unrealistically high compared to the fold-change calculation obtained using the newly defined reference genes (p = 0.03). It was observed that cultures treated with LPS and Poly I:C overexpress both CB receptors compared to untreated cultures, the mean fold change in the normalization for CB1 with CRN was 4.4 and 2.6, with GAPDH it was 3 and 2.4, while with *β-actin* it was 150 and 43, respectively (Fig. 1C). LPS and Poly-I:C stimuli induced a CB2 overexpression that was measured as 14 and 8 with CRN, 2.9 and 2.5 with GAPDH, and 46,000 and 324,500 fold increase with *β-actin* (Fig. 1D).

## 4 Discussion

Odontoblasts are differentiated cells found in the dental pulp that occur in the form of palisades, with prolongations extending along the dentinal tubules. They are the first dental cells to respond to external chemical and physical stimuli, such as changes in temperature and osmolarity, loss of dentinal integrity, and bacterial overgrowth.^10^ Therefore, exploring the different expression profiles of these cells is crucial for understanding their physiological roles in the processes of dental pulp nociception and inflammation.

CB1 and CB2 receptors are G-protein-coupled receptors found in various cell types, including cell membranes, nuclei, and organelles.^11^ They are expressed in dental pulp and odontoblasts in murine models.^12^ CB1 expression has also been reported in human odontoblasts.^13^ Therefore, it is crucial to study the presence of both receptors in human odontoblasts to potentially use them as pharmacological targets for the treatment of dentin sensitivity, dental pain, and inflammation.

To evaluate the expression of CB1 and CB2 receptors in human odontoblasts and dental pulps, reference genes are required. This work aimed to describe a strategy for screening and validating some housekeeping or reference genes for relative quantification of messengers, even during inflammation-mimicking conditions. Thus, we identified a set of reference genes with stable behavior that provides the best transcripts to perform data normalization and evaluation of the expression of the CB receptor transcript of interest in human odontoblasts.

To obtain the best possible reference genes, the requirements for accurate gene expression evaluation involve a rigorous flowchart and validation steps. It is important that candidate genes are chosen from a set of sequencing data previously evaluated in tissues and cells of interest.^14^^,^^15^ Here, gene selection was performed using Genenvestigator, an online tool that helps to identify genes that show high expression stability in a chosen set of conditions.^15^ In this way, researchers can identify the genes that are most stably expressed, among all the genes obtained from microarray data, under desired conditions or experiments. This microarray database (47,000 probe sets on more than 5000 arrays) was systematically annotated and quality-controlled in several organisms. They used the Affymetrix system, which is a highly reproducible microarray system; therefore, all protocols, quality control measures, and data have a high degree of homogeneity.^15^

The second step for reference gene validation was the use of the Bestkeeper tool, which determines the most suitable standards out of ten candidates and combines them into an index.^16^ This index can be compared with ten other target genes to determine whether they are differentially expressed under an applied treatment. According to Pfaffl et al., all data processing is based on crossover points; genes with an SD less than 1, with a higher Pearson correlation coefficient, and with an x-fold less than 2 are considered to be the most stable genes.^16^

The results showed that *β-actin* had the lowest SD; however, it had very high expression levels compared to the target genes. On the other hand, *18S* had the highest expression levels but also one of the largest SD; therefore, it was excluded from relative quantification. This is consistent with other studies that reported that relatively high means of expression are obtained when using ribosomal RNA.^13^ Although *GAPDH* yielded the lowest SD, it had higher expression levels than those of the target genes, although it was the commonly used reference gene that showed appropriate results. The gene that showed the best results with a low SD and a similar level of expression to CB1 and CB2 was *CRN* (Fig. 1B). There was no evidence of its prior use as a reference gene.

The results of the validation of genes using the Bestkeeper tool were in agreement with the first data analysis. *CRN* was the gene that showed the highest correlation coefficient among the most stable genes, which reinforces the idea that it is a suitable gene for use in relative qPCR quantification in human odontoblast cultures.

The relative quantification Schefe's method, which uses the calculated efficiency of the PCR reaction, yields the fold-change number of a gene. We used the reference genes with the best expected performance (*LMX*, *FAM2*, *GAPDH*, *CRN*, *β-actin*) to normalize the CB1 and CB2 transcripts, and found significant differences in their expression levels after the use of *β-actin*. In all cases, it was observed that under inflammatory conditions, odontoblasts overexpressed both receptors, especially CB2, which plays an essential role in the regulation of inflammation (Fig. 1C–D).^17^, ^18^, ^19^ This analysis demonstrates that for a reference gene, it is not enough to have a low variability in expression; it should also have a similar level of expression (Cq) to the target gene. This explains why *β-actin* has been discarded as a reference gene in many studies.^7^^,^^20^ *GAPDH* showed no statistically significant differences compared to the other genes. The relative quantification results confirmed that the best gene for normalization was *CRN*.

For the best results, it is recommended to normalize with at least two reference genes,^2^ and in human odontoblasts, *GAPDH*, *LMX*, or *FAM2* can be used. The geometric mean of these quantifications would yield adequate results to evaluate the expression levels of CB receptors. For the evaluation of another gene of interest in odontoblasts, the same dataset can be used, and the appropriate reference gene should be chosen considering the expression levels of the target gene.

The use of commonly used reference genes can lead to significant distortion in the expression data,^2^ even in odontoblasts. Therefore, it is important to perform the validation of reference genes before evaluating the expression levels of a gene in every biological context, as there are no genes that have universal stability in expression.^15^

## 5 Conclusions

The results of this study showed that the expression levels of internal control genes always vary; therefore, it is necessary to validate the reference genes prior to their use in RT-qPCR normalization in human odontoblasts.

The different data analyzes revealed that the most stable gene with the most suitable expression profile for the relative quantification of CB1 and CB2 transcripts under the evaluated conditions was *CRN*.

Bioinformatics tools aid in the selection of the best reference genes for the sensitive evaluation of gene expression of the molecules of interest, which should be confirmed experimentally. The strategy for the search and verification of reference genes proposed in this study facilitated the determination of CB receptor expression in human odontoblasts with high accuracy and reliability.

## Funding

This research was partially funded by 10.13039/501100002753Biominerales Pharma (Bogota) and Centro de Investigación y Extensión (Facultad de Odontología, Universidad Nacional de Colombia, Bogota), Code: 201010026415.

## Declaration of competing interest

The authors declare that they have no known competing financial interests or personal relationships that could have appeared to influence the work reported in this paper.
